# An Analysis of Percutaneous Nephrolithotomy (PCNL) Performed at the Institute of Kidney Disease, Pakistan: Stone Clearance and Complications

**DOI:** 10.7759/cureus.75430

**Published:** 2024-12-09

**Authors:** Muhammad Ashhad Ullah Khalil, Yassar Hussain Patujo, Farman Ullah, Usama Ibrar, Ruqqayia Adil, Qazi Adil Inam, Muhammad Zohaib, Arooj Fatima, Muhammad Asif, Muhammad Afnan

**Affiliations:** 1 Department of Urology, Institute of Kidney Diseases, Hayatabad Medical Complex, Peshawar, PAK; 2 Department of Urology, Chandka Medical College, Shaheed Mohtarma Benazir Bhutto Medical University, Larkana, PAK; 3 Department of Urology, Lady Reading Hospital, Peshawar, PAK; 4 Department of Urology, Sheikh Zayed Hospital, Rahim Yar Khan, PAK; 5 Department of Diagnostic Radiology, National University of Sciences and Technology (NUST) School of Health Sciences, Islamabad, PAK; 6 Department of Urology, Nawaz Sharif Medical College, Gujrat, PAK; 7 Department of Medicine, Combined Military Hospital Kharian Medical College, Kharian, PAK; 8 Department of Cardiovascular Medicine, Khyber Medical Institute of Medical Sciences, Kohat, PAK

**Keywords:** complications, healthcare outcomes, percutaneous nephrolithotomy, renal stones, stone clearance

## Abstract

Background: Percutaneous nephrolithotomy (PCNL) is the preferred treatment for large renal stones, yet variability in outcomes arises from patient-specific factors and institutional practices. Understanding complications and predictors of success is essential to improving procedural efficacy.

Objective: This study aimed to evaluate stone clearance rates, complications classified using the Clavien-Dindo system, and predictors of PCNL outcomes, with a focus on improving lower calyx stone clearance.

Methodology: A retrospective analysis was conducted on 422 PCNL procedures performed from July 2021 to December 2023 at the Institute of Kidney Disease, Hayatabad Medical Complex, Peshawar, Pakistan. Patient demographics, stone characteristics, and postoperative outcomes were analyzed. Stone clearance rates were calculated, and complications were categorized by severity. Associations between patient and stone characteristics and outcomes were tested using Chi-square analysis, with significance set at p < 0.05.

Results: A total of 422 PCNL cases were analyzed, with an overall stone-free rate of 75.82% (320/422). The average patient age was 45.39 ± 12.43 years, with male patients comprising 56.87% of the cohort and a mean BMI of 27.82 ± 4.21 kg/m². Hypertension (21.43%), diabetes mellitus (18.95%), and chronic kidney disease (7.11%) were notable comorbidities. Stones averaged 3.21 ± 1.13 cm in size, predominantly composed of calcium oxalate (35.55%), and were most frequently located in the upper calyx (28.44%). Postoperative outcomes revealed a 21.33% complication rate, with 16.59% classified as minor (Clavien-Dindo Grades I-II) and 4.74% as major (Grades III-IV). Follow-up data showed that 11.83% of patients had residual stone fragments, and 9.48% experienced delayed complications, primarily minor. Stone size and location significantly influenced outcomes, with clearance rates of 80.00% for 2-3 cm stones versus 65.57% for stones >3 cm (p = 0.155). Lower calyx stones had markedly reduced clearance rates (50.00%, p < 0.001) compared to upper (75.00%) and mid-calyx (70.00%) stones, while stones in multiple locations also showed poor clearance (57.69%, p = 0.001). Key predictors of complications included higher BMI and prolonged operative times, emphasizing the need for tailored approaches, technical refinements, and institutional audits to optimize outcomes for anatomically or clinically complex cases.

Conclusion: This study highlights the high efficacy of PCNL in managing large renal stones, achieving a stone-free rate of 75.82%. However, the findings underscore the significant challenges associated with lower calyx stones and larger stone sizes, both linked to reduced clearance rates. The Clavien-Dindo classification of complications shows that while minor complications are more frequent, major complications remain a concern, especially in patients with elevated BMI or prolonged operative times. To optimize outcomes, this study recommends refining surgical techniques and adopting technological advancements to improve lower calyx clearance, implementing institutional audits to standardize practices, and personalizing treatment strategies through preoperative risk stratification. Future multicenter prospective studies are needed to validate these findings, further explore predictors of outcomes, and develop comprehensive care protocols addressing anatomical and patient-specific challenges in PCNL procedures.

## Introduction

Percutaneous nephrolithotomy (PCNL) is a major intervention for big renal calculi, especially those greater than 2 cm, and stones resistant to retrograde intrarenal surgery or extracorporeal shock wave lithotripsy [1,2]. In this minimally invasive surgery, a percutaneous route is created through the skin and kidney in order to fragment and remove calculi [3]. Nephroscopes and other devices may then be inserted into this tract. PCNL is a technically demanding procedure that needs careful patient selection, highly competent surgical teams, and sufficient hospital infrastructure to maximize results and reduce problems [4]. The size, location, and makeup of the stones, in addition to patient variables including age, comorbidities, and anatomical variances, all have an impact on the procedure's effectiveness and safety [5].

Recent developments in PCNL technology and procedures such as the use of upgraded lithotripsy devices, smaller tools, and better imaging modalities have greatly raised success rates while reducing damage to renal tissues [6]. Better patient outcomes and quicker recovery are made possible by these advancements, which also enable more accurate stone targeting, shorter surgical timeframes, and narrower access tracts [7]. Even with these advancements, PCNL is still linked to a number of possible side effects. These include postoperative complications like fever, sepsis, or the development of arteriovenous fistulas, as well as intraoperative hazards like bleeding, which may need transfusions, infection, and damage to nearby organs like the gut or pleura [8,9]. Furthermore, insufficient stone removal may require further treatments to remove any remaining stone pieces, which would increase patient morbidity and medical expenses [10]. Consequently, even though PCNL has developed into a very successful therapy for big renal stones, limiting problems and maximizing results still depend on careful patient selection, expert surgical technique, and suitable postoperative care [11].

There are still a lot of unanswered questions about how institution-specific characteristics such as local procedures, surgical skills, and patient demographics impact complication rates and stone removal, despite the wealth of research on PCNL outcomes. While a large number of studies have evaluated PCNL results in a general sense, few have concentrated on data at the institutional level, which might be crucial for creating specialized plans to enhance procedural outcomes and patient safety. By offering a detailed examination of PCNL outcomes particular to the Institute of Kidney Disease, Peshawar, Pakistan, and by offering novel insights into the impact of patient characteristics and institutional procedures on stone clearance and complication rates, this study seeks to close this knowledge gap. Through knowledge of these variables in a specialized healthcare environment, our study aims to provide guidance for clinical and procedural modifications that might improve PCNL efficacy and lower patient risk.

Research objective

This research used a retrospective analysis of clinical results and procedural experiences to evaluate the stone clearance rate and related complications of PCNL done at the Institute of Kidney Disease.

## Materials and methods

This retrospective observational research was conducted at the Institute of Kidney Diseases, Hayatabad Medical Complex, Peshawar, Pakistan. The study examined clinical data from patients treated for renal stones with PCNL over a period of one and a half years, from July 2021 to December 2023.

Inclusion and exclusion criteria

Eligible participants were adults aged 18-70 years who underwent PCNL for kidney stones larger than 2 cm or those resistant to extracorporeal shock wave lithotripsy. Complete medical records, including preoperative imaging, postoperative assessments, and follow-up data were required. Exclusion criteria included patients with incomplete records, congenital kidney anomalies, recent kidney infections, or previous PCNL within six months to avoid confounding from repeat procedures.

Sample size

The sample size was determined using a random stratification convenience sampling technique to ensure a representative cohort of patients undergoing PCNL [12]. This method allowed for the inclusion of patients across different age groups and clinical profiles, ensuring diversity in the sample. Based on an expected outcome rate of 50% for maximum sample size efficiency, the target was set at approximately 384 participants. To account for potential dropouts or incomplete data, an additional 10% buffer was added, leading to a final target of around 422 patients. This approach aimed to maintain robust and reliable statistical analysis despite potential attrition.

Data collection

Retrospective data collection was done from patient medical records; information was gathered on demographics, coexisting conditions, stone features (size, location, composition), and PCNL treatment specifics (method, operating time, number of tracts utilized). Postoperative stone-free status and any complications classified as mild or serious according to the Clavien-Dindo classification were among the outcomes assessed [13]. For up to three months after the treatment, follow-up information was collected to evaluate any postponed problems and leftover stone particles.

Statistical analysis

IBM SPSS Statistics for Windows, Version 26.0 (Released 2016; IBM Corp., Armonk, New York, United States) was used to analyze the data. For continuous variables, descriptive statistics such as mean and standard deviation (SD) were used; for categorical variables, frequencies and percentages were given. The percentage of patients who became stone-free was used to compute the stone clearance rate. For categorical data, chi-square tests were used to examine the relationship between stone clearance and characteristics such as stone size and position; p-values less than 0.05 were deemed statistically significant.

Ethical approval

The Institutional Review Board of Hayatabad Medical Complex Peshawar granted ethical clearance for this research (approval number: 823/OD/HMC/2021). Since this was a retrospective study using de-identified data, the IRB waived the need for patient permission. Confidentiality was maintained for all data handling in accordance with institutional policies and ethical standards.

## Results

Patient demographics, concomitant diseases, stone features, and specifics of the PCNL technique are shown in Table 1. The average age of subjects was 45.39 ± 12.43 years, with 240 male (56.87%) and 182 female (43.13%) patients. The average BMI was 27.82 ± 4.21 kg/m². Comorbidities included diabetes mellitus (n = 80, 18.95%), hypertension (n = 90, 21.43%), and chronic kidney disease (n = 30, 7.11%). The average stone size was 3.21 ± 1.13 cm. The majority of stones were composed of calcium oxalate (n = 150, 35.55%) and were most commonly located in the upper calyx (n = 120, 28.44%). Among the cases, standard PCNL was performed in 312 patients (73.93%), while mini-PCNL was performed in 110 patients (26.07%). The average operative time was 85.42 ± 20.31 minutes, and an average of 1.56 ± 0.68 tracts were used per procedure.

**Table 1:** Patient Demographics, Comorbid Conditions, Stone Characteristics, and PCNL Procedure Details Data given as frequency (percentage), except where marked as mean ± SD PCNL: percutaneous nephrolithotomy

Variable		Frequency (percentage)
Age (years)	Mean ± SD	45.39 ± 12.43
Gender	Male	240 (56.87)
	Female	182 (43.13)
Body Mass Index (kg/m²)	Mean ± SD	27.82 ± 4.21
Comorbid Conditions	Diabetes Mellitus	80 (18.95)
	Hypertension	90 (21.43)
	Chronic Kidney Disease	30 (7.11)
	Other	50 (11.85)
Stone Size (cm)	Mean ± SD	3.21 ± 1.13
Location	Upper Calyx	120 (28.44)
	Mid Calyx	100 (23.70)
	Lower Calyx	80 (18.96)
	Pelvis	70 (16.59)
	Multiple Locations	52 (12.32)
Composition	Calcium Oxalate	150 (35.55)
	Struvite	70 (16.59)
	Uric Acid	60 (14.22)
	Cystine	30 (7.11)
	Mixed	112 (26.54)
PCNL Procedure	Standard PCNL	312 (73.93)
	Mini-PCNL	110 (26.07)
Operative Time (minutes)	Mean ± SD	85.42 ± 20.31
Number of Tracts Used	Mean ± SD	1.56 ± 0.68

The postoperative results after PCNL are presented in Table 2. A total of 320 patients (75.82%) were found to be stone-free, while 102 patients (24.18%) still had stones. Ninety-three individuals (21.33%) had difficulties; the other 332 patients (78.67%) had no issues at all. Clavien-Dindo Grade I (9.48%) and Grade II (7.11%) were minor problems. Significant problems were less frequent; of the patients, 2.38% had Clavien-Dindo Grade IIIa, 1.19% had Grade IIIb, and 1.19% had Grade IV.

**Table 2:** Postoperative Outcomes: Stone-Free Status and Complications

Outcome		Frequency (Percentage)
Postoperative Stone-Free Status	Stone-Free	320 (75.82)
	Residual Stones	102 (24.18)
Complications	No Complications	332 (78.67)
	Total Complications	90 (21.33)
Minor Complications	Clavien-Dindo Grade I	40 (9.48)
	Clavien-Dindo Grade II	30 (7.11)
Major Complications	Clavien-Dindo Grade IIIa	10 (2.38)
	Clavien-Dindo Grade IIIb	5 (1.19)
	Clavien-Dindo Grade IV	5 (1.19)

Table 3 presents follow-up results for a period of three months after PCNL. In 50 patients (11.83%), there were residual stone particles; in 372 patients (88.17%), there were none. Of the patients, 382 (90.52%) had no issues, while 40 (9.48%) had delayed complications. Clavien-Dindo Grade I occurred in 17 patients (4.02%) and Grade II in 13 individuals (3.08%) as minor problems. Five patients (1.19%) with Clavien-Dindo Grade IIIa, three patients (0.71%) with Grade IIIb, and two patients (0.47%) with Grade IV had major problems.

**Table 3:** Follow-Up Outcomes (Up to Three Months Post Procedure)

Follow-Up Outcome		Frequency (Percentage)
Residual Stone Fragments	Present	50 (11.83)
	Absent	372 (88.17)
Delayed Complications	No Complications	382 (90.52)
	Total Complications	40 (9.48)
Minor Complications	Clavien-Dindo Grade I	17 (4.02)
	Clavien-Dindo Grade II	13 (3.08)
Major Complications	Clavien-Dindo Grade IIIa	5 (1.19)
	Clavien-Dindo Grade IIIb	3 (0.71)
	Clavien-Dindo Grade IV	2 (0.47)

The stone clearance rates according to stone size and position are analyzed in Table 4. A total of 240 patients (80.00%) were stone-free for stones of 2-3 cm, whereas 60 patients (20.00%) still had stones (p = 0.155). Of the patients with stones greater than 3 cm, 42 patients (34.43%) had residual stones and 80 patients (65.57%) were stone-free. The position of the stones was shown to be substantially linked with residual stones (p < 0.001); the clearance rate of upper calyx stones was 75.0% (p = 0.820), that of mid calyx stones was 70.0% (p = 0.133), and lower calyx stones was 50.0%. Multiple places revealed 57.69% clearance (p = 0.001), whereas the pelvic stones had a 71.42% clearance rate (p = 0.408).

**Table 4:** Analysis of Stone Clearance Rates by Stone Size and Location Statistical significance, determined using the Chi-square test (p < 0.05), is presented for stone size, location (stone-free vs. residual status), and composition (calcium oxalate, struvite, uric acid, cystine, mixed) to analyze clearance rates.

Variable	Sub-categories	Stone-Free Status, n (%)	Residual Stones, n (%)	Chi-Square Value	p-value
Stone Size (cm)	2-3 cm	240 (80.00%)	60 (20.00%)	2.023	0.155
	> 3 cm	80 (65.57%)	42 (34.43%)		
Stone Location	Upper Calyx	90 (75.00%)	30 (25.00%)	0.051	0.82
	Mid Calyx	70 (70.00%)	30 (30.00%)	2.26	0.133
	Lower Calyx	40 (50.00%)	40 (50.00%)	17.38	< 0.001
	Pelvis	50 (71.42%)	20 (28.58%)	0.686	0.408
	Multiple Locations	30 (57.69%)	22 (42.31%)	10.16	0.001
Stone Composition	Calcium Oxalate	150 (75.00%)	50 (25.00%)	1.56	0.251
	Struvite	70 (80.00%)	20 (20.00%)	0.28	0.598
	Uric Acid	60 (80.00%)	15 (20.00%)	1.23	0.268
	Cystine	30 (85.00%)	5 (15.00%)	0.87	0.351
	Mixed	112 (70.00%)	48 (30.00%)	2.48	0.212

## Discussion

In this study, 24.18% of patients still had stones following PCNL, for a total stone-free percentage of 75.82%. These results are in line with other studies, which note that, depending on variables such as stone size, position, and surgical skill, stone-free rates after PCNL may range from 70% to 90% [6,14]. In line with our study's 80.0% stone-free rate for this stone size, a study by Rizvi et al. showed a comparable overall success rate of 80% for patients receiving PCNL for big renal calculi, especially for stones between 2-3 cm in size [15]. Patients with stones larger than 3 cm had a lower clearance rate (65.57%). This is in line with other research that shows larger stones are more difficult to completely remove because they frequently involve more complex renal anatomy and require multiple tracts [16]. The lower calyx had a much lower stone-free percentage (50.0%) according to our data than other regions such as the upper calyx (75.0%) and renal pelvis (71.42%). This is in line with other studies showing that anatomical obstacles and gravitational forces make it harder for fragments to pass through lower calyx stones effectively [17].

Major problems (Grades IIIa and above) were less common, occurring in just 4.76% of patients, whereas mild difficulties (Clavien-Dindand II) accounted for 16.66% of cases. The overall complication rate was 21.33%. These percentages are comparable to earlier research, which found that 28% of problems were of a mild nature overall [18]. The relative rarity of major problems in our study group, such as bleeding recurrence or severe kidney damage, underscores the safety and effectiveness of PCNL when carried out by skilled surgical teams.

At three months following the treatment, follow-up data revealed that 88.17% of patients had no remaining stone pieces, demonstrating long-lasting stone removal. 9.48% of patients had delayed problems, with mild complications (4.02%) more prevalent than significant complications (1.19%). This follow-up result is in line with previous research findings, which showed that delayed problems after PCNL usually varied from 5% to 12% [19]. The low rate of significant delayed problems demonstrates how well postoperative monitoring and follow-up treatment may reduce long-term adverse outcomes.

Study strengths and limitations

This study's large sample size of 422 patients and detailed data collection on demographics, stone characteristics, and procedural details represent significant strengths, enabling a robust evaluation of PCNL outcomes. Although conducted at a single institute, the Institute of Kidney Disease serves as a referral center for patients across Khyber Pakhtunkhwa (KPK), Pakistan, ensuring that the study includes a broad and diverse participant base. The retrospective design provides valuable real-world insights into clinical practices and outcomes, contributing practical knowledge to the existing body of research. To minimize biases inherent in retrospective studies, rigorous inclusion and exclusion criteria were applied, and standardized data extraction protocols were used to enhance the accuracy and reliability of postoperative assessments. However, limitations such as retrospective design and reliance on existing records persist. To overcome these gaps, future research should focus on prospective, multicenter studies involving diverse populations and clinical environments, enhancing the generalizability and applicability of the findings.

## Conclusions

This research highlights the safety and efficacy of PCNL in the management of big renal stones resulting in a remarkable 75.82% stone-free rate and a 21.43% overall complication rate. These findings demonstrate the substantial influence of stone features on treatment effectiveness in addition to reaffirming the vital role that careful patient selection and surgical technique play in maximizing outcomes. The current study adds to our understanding of PCNL by offering insightful information about institutional practices and how they affect procedural efficacy. It also highlights the need for continued improvement of surgical techniques in order to improve patient safety and treatment efficacy in the future.
